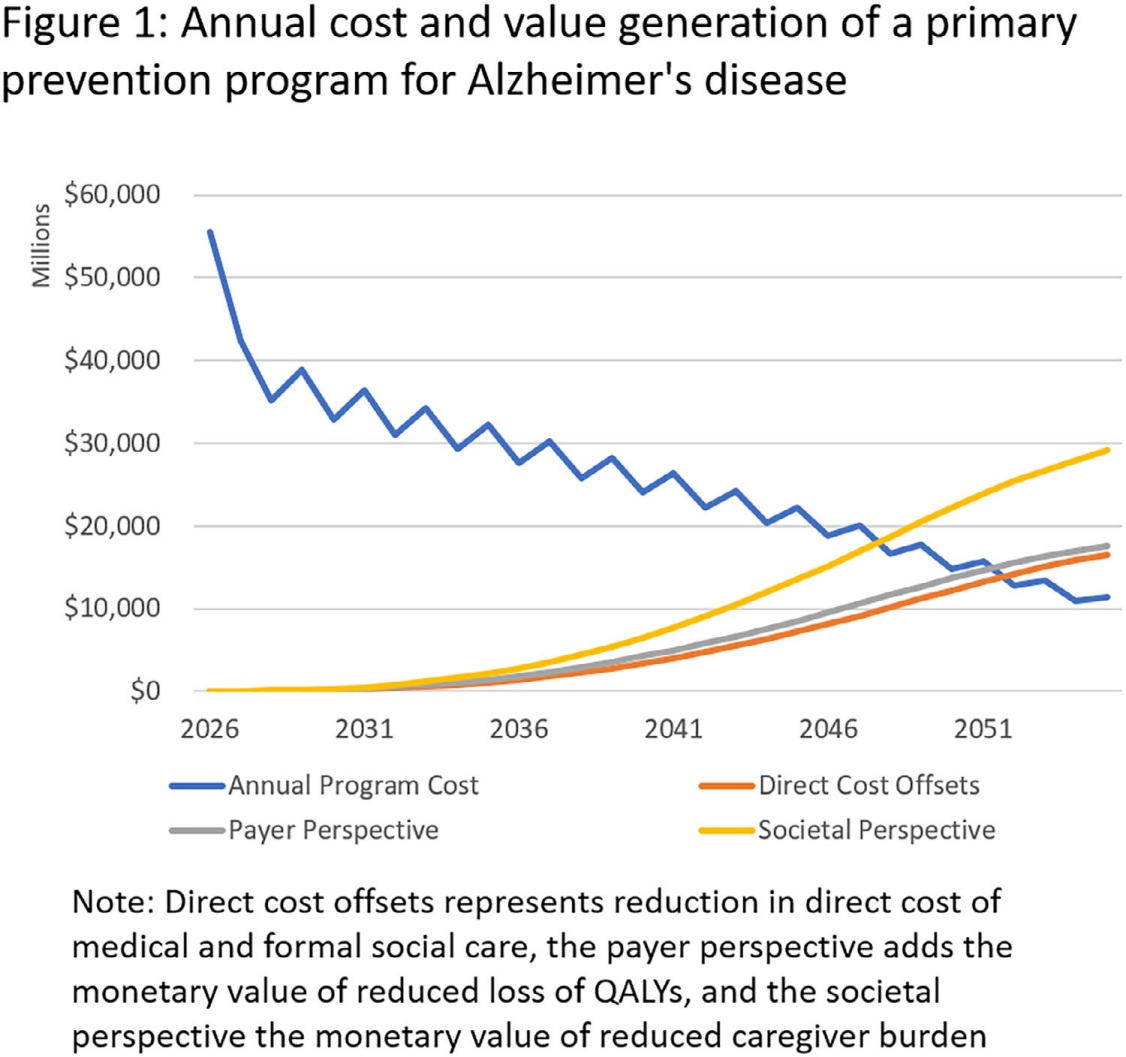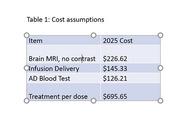# Can primary prevention of Alzheimer's disease be cost‐effective?

**DOI:** 10.1002/alz70860_104588

**Published:** 2025-12-23

**Authors:** Soeren Mattke, Jiahe Chen, Eric M. Reiman

**Affiliations:** ^1^ University of Southern California, Los Angeles, CA, USA; ^2^ University Of Southern California, Los Angeles, CA, USA; ^3^ Banner Alzheimer's Institute, Phoenix, AZ, USA

## Abstract

**Background:**

Pharmacologic treatments for preclinical Alzheimer's disease (PCAD) – the presence of Alzheimer's pathology in cognitively intact individuals ‐ are in advanced clinical trials. Prior research suggested that a screening/treatment program for PCAD could be cost‐effective. An important question is whether preventing the pathology from developing could also be cost‐effective. We are modeling the clinical and economic value of a hypothetical primary prevention program for the US.

**Method:**

We assume that the 2026 cognitively unimpaired cohort aged 50‐70 with at least one APOE4 allele would undergo annual blood testing (sensitivity and specificity of 0.8, 15% indeterminate results). Individuals testing positive would be considered PCAD and exit the model; those with indeterminate results retested the subsequent year and considered positive if still indeterminate, those testing negative would receive a single preventative dose each year until progression to PCAD. We project the progression to PCAD and subsequently to mild cognitive impairment (MCI) over 30 years comparing natural history to a treatment that reduces progression by 0.75. Reduction in cost, valuation of loss of QALYs and caregiver burden from avoided MCI cases were calculated. Costs of tests and treatment are based on 2025 Medicare rates and list prices, and inflated by 3% annually. (Table 1)

**Result:**

82 million individuals would undergo screening in 2026, 12 million be assigned to treatment based on the first and 1.8 million on the second test. Over 30 years, 410,000 cases of MCI (610,000 versus 1.0 million) would be avoided, with a number needed to treat of 37 to prevent one case. Average net program cost per screened individual would be $9,400, compared with $2,000 (39%) reduction in direct medical and social care cost, $290 (31%) reduction of valued QALY loss and $1,400 (39%) reduction in valued caregiver burden. Figure 1 illustrates that the program would become cost effective in 2052 from a payer (cost reduction and QALY gains) and in 2048 from a societal perspective (adding caregiver burden), but not over the full analysis period.

**Conclusion:**

A hypothetical primary prevention program for Alzheimer's disease generates substantial value. Future work will explore under which conditions it is cost‐effective.